# Towards clinical management of traumatic brain injury: a review of models and mechanisms from a biomechanical perspective

**DOI:** 10.1242/dmm.011320

**Published:** 2013-09-12

**Authors:** Dhananjay R. Namjoshi, Craig Good, Wai Hang Cheng, William Panenka, Darrin Richards, Peter A. Cripton, Cheryl L. Wellington

**Affiliations:** 1 Department of Pathology and Laboratory Medicine, University of British Columbia, Vancouver, BC V5Z 4H4, Canada; 2 Collision Analysis Ltd, 43 Skyline Crescent NE, Calgary, AB T2K 5X2, Canada; 3 Department of Psychiatry, University of British Columbia, Vancouver, BC V6T 2A1, Canada; 4 Synaptic Analysis Consulting Group, Inc., 820 W. Broadway, Suite 202, Vancouver, BC V5Z 1J8, Canada; 5 Departments of Mechanical Engineering and Orthopaedics, University of British Columbia, Vancouver, BC V6T 1Z4, Canada

## Abstract

Traumatic brain injury (TBI) is a major worldwide healthcare problem. Despite promising outcomes from many preclinical studies, the failure of several clinical studies to identify effective therapeutic and pharmacological approaches for TBI suggests that methods to improve the translational potential of preclinical studies are highly desirable. Rodent models of TBI are increasingly in demand for preclinical research, particularly for closed head injury (CHI), which mimics the most common type of TBI observed clinically. Although seemingly simple to establish, CHI models are particularly prone to experimental variability. Promisingly, bioengineering-oriented research has advanced our understanding of the nature of the mechanical forces and resulting head and brain motion during TBI. However, many neuroscience-oriented laboratories lack guidance with respect to fundamental biomechanical principles of TBI. Here, we review key historical and current literature that is relevant to the investigation of TBI from clinical, physiological and biomechanical perspectives, and comment on how the current challenges associated with rodent TBI models, particularly those involving CHI, could be improved.

## Introduction

Traumatic brain injury (TBI; see Box 1 for a glossary of terms) is a leading cause of death and disability worldwide, particularly for persons under 45 years of age (Hyder et al., 2007; Mass et al., 2008). In the United States, the overall incidence of TBI is estimated to be 538 per 100,000 individuals, which represents at least 1.7 million new cases per year since 2003 (Gerberding and Binder, 2003; Langlois et al., 2006; Coronado et al., 2010). The rate of TBI is reportedly lower in Europe (235 per 100,000) and Australia (322 per 100,000) (Cassidy et al., 2004; Tagliaferri et al., 2006), although recent epidemiological data suggest a far greater incidence in the latter (749 per 100,000) (Feigin et al., 2013). Mild TBI (mTBI; synonymous with concussion; see below) makes up ∼75% of all TBI, with an estimated cost to the United States of over US$17 billion per year (Gerberding and Binder, 2003). Falls are the most common cause of severe TBI, and motor vehicle accidents (MVAs), being struck by a moving object and colliding with a stationary object are also common causes (Cassidy et al., 2004; Coronado et al., 2010; Roozenbeek et al., 2013). TBI resulting from high-contact sports such as boxing, American football, ice hockey, soccer and rugby account for almost 21% of all head injuries among children and adolescents in the United States (Centers for Disease Control and Prevention, 2007; American Association of Neurological Surgeons, 2011). TBI is also considered a ‘signature injury’ in modern warfare: ∼20% of veterans from the Iraq or Afghanistan wars have experienced a TBI, 80% of which involved blast injury (Taber et al., 2006; Hoge et al., 2008; Elder and Cristian, 2009). The high incidence of TBI in young people has profound socioeconomic consequences owing to loss of productive years to death and disability. Furthermore, the growing awareness that even mTBI can lead to impaired function (Gavett et al., 2011; Jordan, 2013; Rusnak, 2013; Smith et al., 2013) highlights the urgent need to understand much more about the acute and long-term consequences of brain injury. Many research initiatives are being catalyzed by this tremendous unmet medical need, including major efforts in TBI diagnosis, prognosis and the development of potential interventional, pharmacological and rehabilitative therapeutic strategies. Given that TBI results from mechanical forces acting on the head and brain, the primary purpose of this Review is to inform health and neuroscience-oriented investigators about fundamental biomechanical principles of TBI that can be used to guide both basic and clinical research questions towards the most relevant model systems.

Box 1. **Glossary of terms**

Center of gravity (CG): the point on a body where all the mass can be considered concentrated.
Cerebral blood flow: a measure of blood flow to the brain at a given time.
Concussion: synonymous with mild TBI.
Finite element (FE) model: computational model used to predict stress and strain in a continuous body such as the brain in response to forces and accelerations.
Glasgow coma scale (GCS): neurological scale that provides an objective recording of the state of consciousness of a person. The GCS scales eye, verbal and motor responses, and ranges between 3 (deep coma) to 15 (normal).
Gyrencephalic: folded or convoluted cerebral cortex characterized by gyri (ridges) and sulci (depressions or furrows), which increase the total surface area of the cortex.
Head Injury Criteria (HIC): a measure of the likelihood of head injury arising from an impact.
**HIC_15_**: HIC calculated using a maximum time duration of 15 ms.
Impulsive force: a linear force acting over a short time duration that changes the momentum of a body.
Impulsive moment: a rotational force acting over a short time duration that changes the angular momentum of a body.
Intracranial pressure (ICP): fluid pressure of the cerebrospinal fluid that is measured at the level of the foramen of Monro. The normal values of ICP in infant, child and adult are <7.5 mmHg, <10 mmHg and <15 mmHg, respectively.
Linear acceleration: rate of change of velocity of a body measured in *x*, *y*, *z* coordinates of a Cartesian reference frame. Measured in m/second^2^.
Lissencephalic: less convoluted or smooth cerebral cortex.
Pressure gradient: an engineering measurement of change in pressure per linear length. Pressure gradients result in unbalanced forces acting on objects.
Rotational acceleration: rate of change of angular velocity of a body. Measured in radians/second^2^.
Scaling model: a mathematical model to transfer engineering measurements made with a surrogate model to a real system where measurements cannot be practically taken from the real system.
Shear: shear of the brain is deformation that tends to change the shape of sections or all of the brain. In shear deformation, parallel inner surfaces of brain matter slide past one another.
Strain: engineering measure of deformation of continuous material defined by ratio of change in linear length over original length. Strain represents the amount that a material is stretched.
Stress: engineering measure of force per unit area applied to a continuous material.
Traumatic brain injury (TBI): an alteration in brain function, or other evidence of brain pathology, caused by an external force.
Triaxial accelerometer: a laboratory device for simultaneously measuring linear acceleration in three mutually perpendicular directions.
Wayne State Tolerance Curve (WSTC): describes the relationship between linear head acceleration, duration of acceleration and onset of concussion.

## Pathophysiology, classification and clinical management of TBI

TBI results from mechanical forces such as an object striking the head, or from rapid acceleration and deceleration forces that cause vigorous movement, and thereby tissue deformation, of brain tissue within the skull. These forces produce a primary injury that directly affects neurons, blood vessels and glia, and initiates a plethora of secondary processes that result in complex cellular, inflammatory, neurochemical and metabolic alterations (McIntosh et al., 1996; Blumbergs, 1997; Davis, 2000; Giza and Hovda, 2001; Werner and Engelhard, 2007; McAllister, 2011). These secondary changes develop within hours to weeks after the primary injury and lead to a constellation of events that include axonal injury, impaired cerebral blood flow, metabolic changes, edema, raised intracranial pressure (ICP), increased blood-brain barrier (BBB) permeability, calcium influx, elevated oxidative stress, free-radical-mediated damage, excitatory neurotransmitter release, inflammation and cell death (McIntosh et al., 1996; Blumbergs, 1997; Davis, 2000; Giza and Hovda, 2001; Werner and Engelhard, 2007; McAllister, 2011) (reviewed by Prins et al., 2013).

Conventional clinical TBI taxonomy divides injury severity into three categories: mild, moderate and severe. Among the most commonly accepted TBI classification systems is the Glasgow Coma Scale (GCS) (Teasdale and Jennett, 1974), which measures a patient’s level of consciousness based on verbal, motor and eye-opening responses after injury. Collectively, these parameters are used to define clinical injury severity. A patient with a GCS score of 3–8 (out of 15) is considered to have sustained a severe TBI, 9–12 is moderate, and >12 mild (Teasdale and Jennett, 1974; Teasdale and Jennett, 1976). The prognostic ability of this system is limited and as such other descriptors have been added. In the most recent iteration of the widely adopted Departments of Defense and Veteran Affairs definition of brain injury severity, mTBI is further denoted by the presence of post-traumatic amnesia (PTA) lasting less than 24 hours and a loss of consciousness (LOC) of less than 30 minutes. Similarly, to meet moderate TBI criteria, the GCS score must be between 9 and 12, PTA must not exceed 1 week and LOC must not last longer than 24 hours (U.S. Departments of Defense and Veterans Affairs, 2008).

Compared with moderate and severe TBI, the definition of mTBI is in a period of rapid evolution. The American Congress of Rehabilitation Medicine defined mTBI as ‘traumatically induced physiological disruption of brain function, as manifested by at least one of the following: (1) any period of loss of consciousness; (2) any loss of memory for events immediately before or after the accident; (3) any alteration in mental state at the time of the accident; (4) focal neurological deficit(s) that may or may not be transient; but where the severity of injury does not exceed the following: (a) loss of consciousness of approximately 30 minutes or less; (b) after 30 minutes, an initial GCS of 13–15; and (c) post-traumatic amnesia not greater than 24 hours’ (Kay et al., 1993). Later, the WHO Collaborating Centre Task Force on Mild Traumatic Brain Injury defined mTBI as ‘an acute brain injury resulting from mechanical energy to the head from external physical forces. Operational criteria for clinical identification include: (1) one or more of the following: confusion or disorientation, loss of consciousness for 30 minutes or less, post-traumatic amnesia for less than 24 hours, and/or other transient neurological abnormalities such as focal signs, seizure, and intracranial lesion not requiring surgery; (2) GCS score of 13–15 after 30 minutes post-injury or later upon presentation for healthcare. These manifestations of mTBI must not be due to drugs, alcohol, medications, caused by other injuries or treatment for other injuries (e.g. systemic injuries, facial injuries or intubation), caused by other problems (e.g. psychological trauma, language barrier or coexisting medical conditions) or caused by penetrating craniocerebral injury’ (Carroll et al., 2004).

Various refinements have been proposed to assist in the subclassification of mTBI. For example, a complicated mTBI is distinguished from an uncomplicated mTBI by the presence of associated intracranial imaging abnormalities consistent with the trauma (Williams et al., 1990). Attempts to further subdivide mTBI based on clinical symptoms have also been made, with varying success. For example, the American Academy of Neurology severity of concussion scale divided mTBI into multiple subcategories (e.g. concussion grades 1, 2, 3, etc.) but, in a recent major revision endorsed by multiple sporting bodies and physician groups, these finer distinctions have now been dropped owing to a lack of prognostic utility (Giza et al., 2013).

A recent review of 100 randomized clinical trials (RCTs) examined the efficacy of interdisciplinary interventions of 55 acute-phase and 45 post-acute-phase trials (Lu et al., 2012). Acute-phase trials were defined as interventions that occurred within 24 hours of TBI. These studies largely focus on patient stabilization and minimizing secondary injury, and employ standard outcomes comprised of GCS scores and mortality. By comparison, post-acute trials can be very heterogeneous in design. The post-acute trials reviewed were initiated from days to years post-TBI and used a wide variety of outcomes, including cognitive, neuropsychological and quality-of-life measures (Lu et al., 2012).

In the acute phase, only 15 of 55 trials showed a positive treatment effect and, of these, 11 evaluated non-pharmacological interventions. Furthermore, many interventions that were tested across multiple trials led to mixed results. For example, decompressive craniotomy, hyperosmotic therapy and hypothermia have shown inconsistent effects, with studies reporting positive (Cruz et al., 2001; Cruz et al., 2002; Zhi et al., 2003; Jiang et al., 2005; Qiu et al., 2005; Jiang et al., 2006; Qiu et al., 2007), negative (Cooper et al., 2011) or no (Smith et al., 1986; Marion et al., 1997; Shiozaki et al., 2001; Clifton et al., 2002; Lü et al., 2003; Cooper et al., 2004; Harris et al., 2009; Clifton et al., 2011) effect on outcomes. A single trial of hyperventilation to reduce ICP also revealed an adverse effect (Muizelaar et al., 1991). Other non-pharmacological interventions that have shown benefit include early nutritional supplementation with zinc within 48 hours or total parenteral nutrition within 72 hours post-TBI (Young et al., 1996). Lastly, a pre-hospital rapid sequence intubation trial also yielded positive results (Bernard et al., 2010).

The 32 acute-phase pharmacological trials reviewed were grouped according to the targeted treatment mechanism. Anti-excitotoxic agents, insulin, magnesium, corticosteroids and drugs targeting lipid peroxidation or free radical damage either failed to show a positive treatment effect or led to adverse effects. Importantly, only 4 of 32 pharmacological acute-phase RCTs for TBI showed positive treatment effects, including one involving the psychostimulant methylphenidate (Ritalin) (Moein et al., 2006), two RCTs for progesterone (Wright et al., 2007; Xiao et al., 2008) and one RCT for the calcium channel blocker nimopidine (Harders et al., 1996).

Of the 45 post-acute-phase RCTs reviewed, 24 evaluated cognitive rehabilitation approaches and of these 22 showed positive treatment effects. The approaches used included comprehensive interdisciplinary rehabilitation, cognitive/academic exercises and communication skill training, compensatory techniques and computer-assisted training, educational intervention, psychotherapy, and behavior modification. Six of eight trials using physical rehabilitation also showed positive treatment effects, and nutrition and acupuncture were found to be beneficial in the single trials conducted thus far. Potential TBI pharmacotherapies were tested in 11 post-acute RCTs, with positive treatment effects reported in six studies, including for methylphenidate (Whyte et al., 2004; Willmott and Ponsford, 2009), CDP-choline (Calatayud Maldonado et al., 1991) and pyritinol (Kitamura, 1981). A trial of phenytoin and carbamazepine was negative (Smith et al., 1994), and sertraline, carbamazepine, rivsatigmine and modafinil were found to have no significant treatment effects (Banos et al., 2010; Jha et al., 2008; Novack et al., 2009; Tenovuo et al., 2009).

A number of RCTs in TBI have been completed this past year. Highlights include a Phase III randomized trial of hypothermia in children, termed the ‘Cool Kids’ study, which recently reported that 48 hours of hypothermia followed by rewarming does not reduce mortality or positively affect recovery from pediatric brain injury (Adelson et al., 2013). An intervention trial randomizing high-risk post-concussion syndrome patients to an early visit to a physician similarly was negative, with similar outcomes to the treatment as the control group (Matuseviciene et al., 2013). Chesnut et al. recently reported on their much anticipated trial of continuous invasive ICP monitoring following severe traumatic brain injury, showing no advantage over imaging and clinical examination alone (Chesnut et al., 2012). Citocoline, which demonstrated promise in earlier trials as a neuroprotective agent, failed in a subsequent Phase 3 trial (Zafonte et al., 2012). A trial of 30 hyperbaric oxygen therapy sessions, performed on military personnel with mTBI, did not demonstrate a significant effect (Wolf et al., 2012). Finally, in perhaps the most influential trial of this past year, amantadine, given for 4 weeks to TBI patients who were minimally conscious or in a vegetative state at 4 to 16 weeks following TBI, showed a highly significant effect on accelerating the pace of functional recovery (Giacino et al., 2012).

The emerging consensus from these 100 RCTs conducted on TBI subjects is that early intervention in the acute phase and comprehensive rehabilitation approaches in the post-acute phase provide the most beneficial outcomes. What is far less clear is whether a deeper understanding of the pathways involved in TBI pathogenesis might eventually offer effective pharmacological therapies. A crucial requirement for any drug discovery program is the availability of preclinical models that are as biofidelic to the human condition as possible. With respect to TBI, however, a working knowledge about neurophysiology and biomechanics is necessary to select the most appropriate model system to address the experimental question being considered.

## Experimental models of TBI

An increasingly wide variety of experimental animal models are available to investigate secondary injury processes in TBI and to provide preclinical data for candidate therapeutic approaches. However, because no single model can reproduce the complexity of TBI pathology observed in humans, several large and small animal models have been developed to mimic particular components, and it is important to understand the validity and limitations of each model.

Large animal models (e.g. non-human primates, pigs, dogs, sheep, cats) are useful for investigating questions related to the response of the gyrencephalic brain (a brain in which the cerebral cortex is convoluted, such as that of humans) to injury, and might be particularly applicable for advanced preclinical evaluation of therapeutic agents before introduction into humans (Kwon et al., 2010). However, the high cost of rearing large animals and the considerable ethical concerns associated with their use limits their wide adoption into preclinical studies. In contrast, rodent (e.g. rat and mouse) models have emerged as the most commonly used animal models in TBI research because they are widely available, cost-effective, amenable to many behavioral, physiological and drug discovery evaluations, and, particularly in the case of mice, offer a wide variety of genetically modified strains. As with other research involving animal models, it is important to note that ethical considerations are pertinent to this field (Levy, 2012). All federal and institutional animal care committees (such as the Institutional Animal Care and Use Committee in the United States and the Canadian Council on Animal Care in Canada) mandate that experimental procedures using animal models require ethical approval according to best-practice guidelines at the national and institutional levels. These include adequate anesthesia, detailed post-procedure monitoring, and clearly defined mortality and humane end-point limits.

In the following sections, we briefly summarize the most commonly used and established experimental models of TBI (Table 1). Several excellent reviews provide a more detailed overview of the wide variety of *in vivo* animal models of TBI (Lighthall et al., 1989; Povlishock et al., 1994; Finnie, 2001; Cenci et al., 2002; Cernak, 2005; Duhaime, 2006; McCabe et al., 2010; Frink et al., 2011; Marklund and Hillered, 2011; O’Connor et al., 2011; Xiong et al., 2013).

**Table 1.**
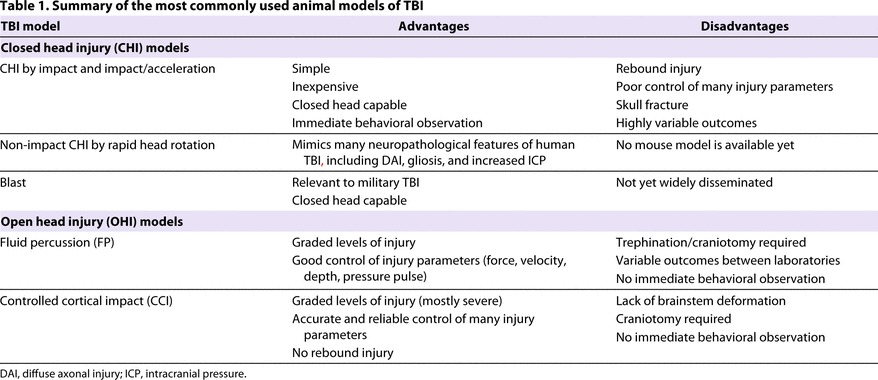
Summary of the most commonly used animal models of TBI

## Open head injury models

In open head injury (OHI) models, which include fluid percussion (FP) and controlled cortical impact (CCI) models, the mechanical force is applied directly to the dura mater (the outermost layer of the meningeal membranes covering brain tissue), which is exposed by a craniotomy. Because the mechanical force is applied directly to the dura mater, little or no head movement is seen in OHI models.

### FP-induced TBI

FP models generate brain injury by rapidly injecting fluid onto the intact dura mater through a craniotomy. Typically, fluid is injected downwards using a midline craniotomy over the sagittal suture midway between the skull anatomical landmarks of bregma and lambda, or sideways using a craniotomy positioned laterally over the parietal cortex (Gurdjian et al., 1966; Thompson et al., 2005; Alder et al., 2011). Fluid propulsion is driven by dropping a pendulum onto a fluid reservoir, and pulse pressure, load duration and pulse velocity are reported. The pressure pulse of the fluid can be varied to produce more- or less-severe injury, thus enabling FP models to mimic a variety of human TBIs. FP induces mixed injury including petechial and subarachnoid hemorrhage, vascular damage at the gray-white matter interface, diffuse axonal injury (DAI), focal necrosis, and cell loss (Povlishock and Kontos, 1985; McIntosh et al., 1987; Dixon et al., 1988; McIntosh et al., 1989; Wang et al., 1997; Thompson et al., 2005). Biomechanical studies of FP models have mainly concentrated on characterizing the input parameters such as volume loading, pressure peaks and rate of fluid flow (Stalhammar et al., 1987). High-speed X-ray imaging of the fluid pulse in rats (Dixon et al., 1988) and ferrets (Lighthall et al., 1989) shows that the complex movement of fluid in the epidural space induces gross movement of brain tissue. Although the biomechanical response of the brain tissue to the fluid movement has been reported (Thibault et al., 1992), less is known about the relationships of brain movement to functional outcomes. Although very widely used, particularly in rats, FP is associated with a high mortality rate because it induces apnea. Also, there is considerable variability of outcomes among different laboratories (Cernak, 2005; Marklund and Hillered, 2011). This variability is hypothesized to be due in part to the surgical precision required to generate a highly reproducible craniotomy position and angle. As a result, use of FP models often requires extensive operator training.

### CCI-induced TBI

CCI models involve a weight-drop device (Feeney et al., 1981) or a pneumatic (Dixon et al., 1991; Smith et al., 1995), electromechanic (Onyszchuk et al., 2007) or electromagnetic (Brody et al., 2007) piston to deliver precisely controllable tissue deformation to an exposed dura mater. This method induces primarily focal damage with extensive cortical loss, hippocampal and thalamic damage, edema, and increased ICP (Saatman et al., 2006; Marklund and Hillered, 2011), and is thereby used primarily to mimic severe TBI with frank tissue destruction. The injury severity is controlled by the impactor size and design, impact velocity, depth, and dwell time of brain compression, which can be adjusted to produce varying degrees of injury with excellent precision and reproducibility. CCI models do not suffer from ‘rebound injury’ typically seen in weight-drop models (see below). On the other hand, the injury induced in the majority of CCI studies destroys large cortical areas, which is observed only in severe human TBI (Marklund and Hillered, 2011). As a result, many CCI-based investigations recapitulate a limited subset of human TBI cases.

## Closed head injury models of TBI

In closed head injury (CHI) TBI models, the injury is induced through the intact skull by direct impact (e.g. dropping a weight on the intact skull or striking the intact skull with a piston), non-impact (e.g. blast) or inertial loading (by rapid rotation of head in the sagittal, coronal or oblique planes). CHI models are characterized by varying degrees of head acceleration.

### CHI induced by impact

In impact CHI, a mechanical force is delivered to the intact skull, most commonly by dropping a weight from a pre-determined height or by using a pneumatically or electromagnetically driven impactor. The force can be directed to the vertex (Tang et al., 1997a; Tang et al., 1997b; DeFord et al., 2002; Zohar et al., 2003; Creeley et al., 2004; Wang et al., 2006), the lateral side (Laurer et al., 2001; Shitaka et al., 2011) or the frontal side (Kilbourne et al., 2009) of the skull.

Weight-drop models that use the gravitational forces of a free-falling weight have been developed for both rats and mice and are among the simplest and most widely used models in TBI research (Marmarou et al., 1994; Chen et al., 1996; Tang et al., 1997a; Tang et al., 1997b; Beni-Adani et al., 2001; DeFord et al., 2002; Pan et al., 2003; Zohar et al., 2003; Ucar et al., 2006; Flierl et al., 2009; Zohar et al., 2011). Injury severity can be adjusted by varying the drop weight or drop height. Methods to dissipate energy across the skull and allow greater skull movement upon impact by using flexible platforms can shift the injury type from a more focal to a more diffuse pattern. In the Marmarou method, for example, the rodent’s head is supported on a thick block of foam or gel that allows partial head acceleration, which causes moderate to severe brain injury with DAI, which is characterized by prominent amyloid precursor protein (APP) immunostaining (Foda and Marmarou, 1994; Marmarou et al., 1994; Viano et al., 2012). Recently, Kane et al. described a new murine CHI model (Kane et al., 2012) characterized by a completely unrestrained head and body. In this model, the animal is supported only by a sheet of aluminum foil suspended over an empty case with thick foam pad at the base. The aluminum foil completely tears off during impact, allowing unrestricted head and body movement of the animal as it falls into the cage (Kane et al., 2012). These authors reported that mild concussive injury could be repeatedly induced, suggesting that this technique could be used for repeated impacts.

Advantages of CHI models include the concordance of the mechanism with the vast majority of human TBI that occur without skull fracture and, relative to FP and CCI, the use of simpler surgical methods that allow rapid behavioral assessment of injury severity. However, weight-drop models pose a number of substantial limitations. For example, high-speed videography shows that many weight-drop models deliver both a primary and a secondary rebound impact to the head, with the rebound injury essentially representing a poorly controlled second impact. In addition, weight-drop models are limited in that the velocity of head impact and head displacement cannot be varied independently as they can be when using an actuator. Furthermore, appropriate release of head constraint to allow human-like head kinematics is challenging with weight-drop models and actuators that move downwards to create impact. A principal caveat of most current CHI animal models is that both the input parameters (e.g. mechanical loading, method of mechanical input, response of the animal’s head to mechanical loading) as well as the cognitive, histological and biochemical end points used can vary considerably among different laboratories, which has challenged the reproducibility of results and, thus, the translational potential of these models. Much more needs to be learned about how these CHI models alter animal head kinematics (e.g. calculation of linear versus angular acceleration, quantification of head acceleration), how head kinematics relate to injury severity and, last but not least, how brain movement relates to head movement and functional, molecular and histological outcomes in the species used.

### Non-impact CHI using blast waves

Improvised explosive devices (IEDs) are a major source of TBI in military personnel. Blasts can produce brain injury through several mechanisms, including the energy of the blast wave itself, the generation of acceleration and deceleration forces, as well as particles that impact the head during the blast. These injuries typically present with diffuse edema accompanied by hyperemia and delayed vasospasm. Models that use compressed air or gas or explosives have been developed to simulate a non-impact blast injury (Rubovitch et al., 2011; Goldstein et al., 2012; Rafaels et al., 2012). The degree of head motion after the blast seems to have a significant effect on behavioral and neuropathological outcomes, as reported in a recent study showing that stabilizing the rodent head during the blast acts as a neuroprotective mechanism (Goldstein et al., 2012).

### Non-impact CHI induced by inertial loading

Many laboratories have developed inertial loading CHI models in which rotational forces cause rapid acceleration of the animal’s head followed by a longer deceleration phase. These models have been developed for larger animals, including non-human primates (Gennarelli et al., 1981; Gennarelli et al., 1982), pigs (Smith et al., 1997; Smith et al., 2000; Browne et al., 2011) and rabbits (Gutierrez et al., 2001; Runnerstam et al., 2001; Hamberger et al., 2003; Krave et al., 2005; Krave et al., 2011), as well as for small animals such as rats (Davidsson and Risling, 2011). In these models, the animal head is often secured onto the mechanical system with a snout clamp or a skull-fixation plate. Linear motion induced by a piston is converted to rotational motion of the device, which in turn results in rotational acceleration of the head, along either the coronal (Gennarelli et al., 1982; Smith et al., 1997; Smith et al., 2000), sagittal (Gennarelli et al., 1982; Gutierrez et al., 2001; Runnerstam et al., 2001; Davidsson and Risling, 2011; Krave et al., 2011), axial (Smith et al., 2000; Browne et al., 2011) or oblique (Gennarelli et al., 1982) plane. The degree of trauma can be adjusted by varying the angle of rotation or pulse duration. Studies using these models have shown that post-traumatic neurological status, such as coma, is related to the energy and form of rotation induced (Gennarelli et al., 1981; Gennarelli et al., 1982; Browne et al., 2011; Krave et al., 2011). These models have also shown that rotational forces can induce pathologies including subarachnoid hemorrhage, increased ICP and a rise in serum levels of S100B, a calcium-binding protein that might be a promising biomarker for TBI (Runnerstam et al., 2001; Davidsson and Risling, 2011). A distinctive neuropathological feature of these models is DAI, as evidenced by the formation of axonal bulbs and APP accumulation (Smith et al., 2000; Hamberger et al., 2003; Davidsson and Risling, 2011). Other microscopic pathologies include gliosis (Smith et al., 1997; Gutierrez et al., 2001), neuronal death (Smith et al., 1997; Runnerstam et al., 2001) and the accumulation of phosphorylated heavy neurofilament subunits at neuronal cell bodies (Smith et al., 1997; Smith et al., 2000; Hamberger et al., 2003).

## Choosing the appropriate TBI model

The choice of the most appropriate preclinical model to use depends on what factors of human injury need to be modeled. FP and CCI models induce highly reproducible injuries but have some disadvantages. First, these models represent only the small minority (0.8–3%) of human injuries that involve penetration of the dura (Masson et al., 2001; Myburgh et al., 2008; Wu et al., 2008). Second, these models are most commonly used to induce severe injury, which is typically observed only in severe human TBI (Marklund and Hillered, 2011). Third, both FP and CCI models require complex surgical methods, including a precisely positioned craniotomy that can reduce brain swelling after injury, much like a decompressive craniotomy that is often used to lower ICP after severe TBI (Zweckberger et al., 2006; De Bonis et al., 2010). Fourth, these methods of injury cause almost no head movement, which is rarely observed in human TBI. Therefore, CHI models that apply impulsive (impact) loads to the head are increasingly attractive for modeling the majority (>95%) of human TBIs that occur without skull fracture or penetration of brain tissue. However, most CHI model systems have substantial caveats, including the high incidence of skull fracture (necessitating euthanasia of the animal) and highly variable outcomes between laboratories. We can gain further insights into the relative strengths and weaknesses of the various rodent injury models if we interpret these models in the context of what is known about the biomechanics of human, large animal and rodent TBI.

## Biomechanical principles of TBI

Biomechanics is the study of a biological system using mechanics. In the context of TBI, this involves the study of motion, e.g. acceleration, and mechanical loads sustained on or applied to an organism’s brain. During head impact, dynamic mechanical forces act on the skull and brain to cause both linear and rotational movement of the head and skull. This in turn leads to deformation and structural damage of brain and cerebrovascular tissues, which triggers a plethora of secondary injury pathways.

**Fig. 1.**
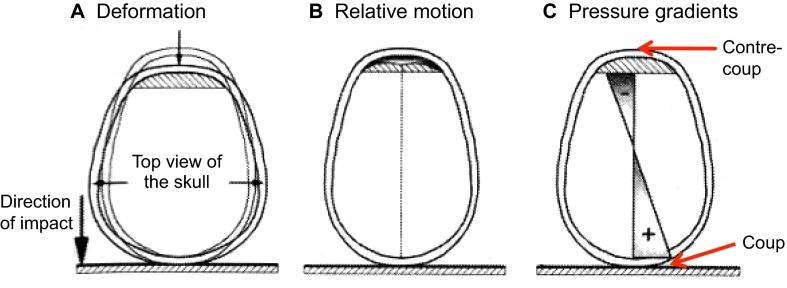
**Basic mechanics of brain and skull deformation during impact.** This example represents a fast-moving head hitting a stationary object. The front of the head is shown to the top of each image. Impact to the back of the skull with the skull moving downwards (large arrow) causes momentary skull deformation (small arrows; A). The skull stops suddenly (in much less than 1 second, i.e. in about 50 ms) and the brain has momentum and attempts to keep moving, causing relative motion of the brain with respect to the skull (B). This motion of the brain sets up positive pressure at the impact (i.e. coup) site and negative pressure opposite the coup site at the contre-coup site (C). Hatched areas in the anterior region of the skull conceptually represent space where the brain has moved away from the skull. Adapted with permission (Schmitt and Niederer, 2004) and outside the scope of the CC-BY license.

Impact TBI in humans can occur under various conditions. One example is when a moving object impacts a slowly moving or stationary head, such as when a vehicle strikes a pedestrian’s head. Conversely, a head moving at a high rate of speed could impact a stationary object, such as when a hockey player slides head first into the boards. These impacts cause intense mechanical loading (i.e. forces applied to the brain), which, despite lasting for only a fraction of a second (<50 ms) (Gurdjian et al., 1966; Ono et al., 1980; Pellman et al., 2003), causes pressure gradients and mechanical strain (i.e. local areas of stretching or compression) to be induced within brain tissue (Meaney and Smith, 2011), as illustrated in Fig. 1. Head impact can result in pure linear (straight line) motion of the head or combined linear and rotational motion in response to impact. Because the head is coupled to the body by the neck, almost any impact to the head will result in a combination of linear and rotational motion (Greaves et al., 2009). In pure linear motion of the head, the pressure gradients and tissue strains described above will occur. In combined rotational and translational motion, the pressure gradients and tissue strains described above will be much larger, because strains from head rotation are superimposed on the pressure-gradient-related strains (Meaney and Smith, 2011).

In contrast to impact TBI, pure blast TBI resulting from an explosive shock wave is non-contact and involves dynamic forces with very short durations on the order of a few microseconds (μs) (Goldstein et al., 2012). Other non-contact TBI mechanisms are hypothesized to occur as a result of ‘inertial’ loading of the head, where torso motion causes the head to move even when no impact forces are received. Theoretical examples include the impact on a football player’s torso from another player during a tackle and the effect of ‘shaken baby’ syndrome. This proposed mechanism of TBI is a matter of some controversy and many investigators have concluded that this does not occur in real-world human TBI (Lau et al., 1989; McLean, 1995; Yoganandan et al., 2009; Meaney and Smith, 2011; Wright et al., 2013) because the duration of inertial head loading in these situations would be too long (Gennarelli, 1993). Most epidemiological studies also conclude that these injuries cannot feasibly occur in adult humans (Lau et al., 1989; McLean, 1995).

TBI can also result from static or near static loads that essentially crush the brain and skull, resulting in direct compression of the brain or contusion injury via bone fragments (Denny-Brown and Russell, 1940; López-Guerrero et al., 2012; Mattei et al., 2012). In these crushing or nutcracker injuries, the head is generally not subjected to the rapid linear or rotational movements that occur during impact injuries.

## Head motion during impact TBI

Head and skull acceleration that occurs during a head impact can be described using three-dimensional linear (translational) and rotational (angular) accelerations. Linear acceleration is defined as the change in velocity over a given time through translational coordinates of the head’s center of gravity (CG), and is usually expressed in units of ‘***g***’ (one ***g*** is the acceleration due to gravity on Earth) or m/second^2^. Rotational acceleration is the change in rotational velocity of the head over a given time and is expressed in units of radians (rad)/second^2^ or degrees/second^2^. One revolution is equal to 360 degrees or 2π radians. Acceleration can be measured using devices called accelerometers.

The relative amounts of linear and rotational head acceleration that result from a particular head impact depend on several factors, including the type of impact force, the direction of the force, the location of the force on the skull, and the material properties of the skull and brain. An impulsive contact force applied to the head is a vector with magnitude and direction. A force that passes through the CG of the head (i.e. aligned with the maxilla) will primarily initiate linear motion of the head during the impact. A force that does not pass through the CG (i.e. impact to the high forehead) will produce an impulsive moment (conceptually a ‘twisting force’) about the CG, which will initiate both linear and rotational acceleration.

There is considerable debate about whether linear (Gurdjian et al., 1955; Haddad et al., 1955; Gurdjian et al., 1961) or rotational (Holbourn, 1943; Gennarelli et al., 1981; Gennarelli and Thibault, 1982; Gennarelli et al., 1982) acceleration is a better predictor of brain injury. Advocates of the rotational acceleration model argue that pure linear impact is rare in the clinical setting and angular acceleration is the principal mechanism underlying brain injury (Holbourn, 1943; Hardy et al., 1994). Notably, the Head Injury Criteria (HIC), which is currently incorporated in vehicle safety standards around the world, takes only linear acceleration into account. The HIC is an analytical expression in which the severity of an impact is calculated based on both the magnitude and duration of the acceleration pulse. In the context of automotive safety testing, the HIC has been credited with considerably reducing the incidence of MVA-related head injuries for over three decades (King et al., 2004). Moreover, recent studies by King et al. contend that helmets significantly reduce linear acceleration without changing rotational acceleration (King et al., 2003), leading these authors to propose that the response of the brain itself (i.e. deformation of the structures of the brain) to mechanical loading might be a better predictor of brain injury than linear or rotational acceleration of the skull (Hardy et al., 2001; King et al., 2003; King et al., 2004; King et al., 2011). Many researchers have proposed metrics that are a combination of both linear and rotational acceleration (and other factors such as HIC and impact force location on the skull) (Gurdjian, 1975; Ono et al., 1980; Pellman et al., 2003; Greenwald et al., 2008; Rowson and Duma, 2013) as the most predictive mechanisms of brain injury (see below).

## Brain motion during impact TBI

Like most soft tissue, the brain has viscoelastic properties with nonlinear mechanical stress-strain responses (LaPlaca et al., 2007). Importantly, it is shear strain – rather than tissue compression, pressure gradients or axonal breakage – that is believed to be the major mechanism underlying most concussion pathology (Meaney and Smith, 2011). A variety of *in vitro* models, typically using flexible supports for neuronal or slice cultures, are available to investigate how specific stretching or twisting motion leads to axonal or cerebrovascular damage (Cullen and LaPlaca, 2006; Alford et al., 2011; Hemphill et al., 2011).

A common question regarding rodent models of TBI is whether the response of the lissencephalic (smooth, non-convoluted) rodent brain to impact, acceleration or blast injury is representative of that of the gyrencephalic human brain, which contains ridges (gyri) and furrows (sulci). For example, cases of chronic traumatic encephalopathy (CTE), which is thought to be caused by repeated concussions, commonly show more extensive pathology in sulcal depths than elsewhere along the cortex, suggesting that shear strain might be maximal at the sulcal depths (Smith et al., 2013). Further work is warranted to investigate the role that these anatomical differences could play in the context of human TBI research. Nevertheless, even if lissencephalic versus gyrencephalic differences are found to result in an inability to reproduce anatomical injury patterns observed in humans, the vast potential of rodent models for studies investigating secondary injury pathways and for drug discovery research is a major incentive to continue use of these models.

## Human-tolerance and related biomechanical studies of TBI

The traumatic injury threshold for brain injury (including mild, moderate or severe TBI) and skull fracture in humans has been investigated using several approaches, including experimental animal models, physical brain surrogate models, and studies in athletes (most commonly football and hockey) who experience frequent head impacts and concussion. Computational models have also been used, including purely analytical (i.e. mathematical equations) and finite element (FE) models. In FE models, the structures of interest, such as the brain, meninges and skull, are built by separating three-dimensional models of the structures using tens of thousands or millions of discrete elements. In this way the stresses in highly irregular shapes composed of complex materials (like gray and white matter in the brain) can be calculated by solving the relatively simple stress state in each of the small elements.

**Fig. 2.**
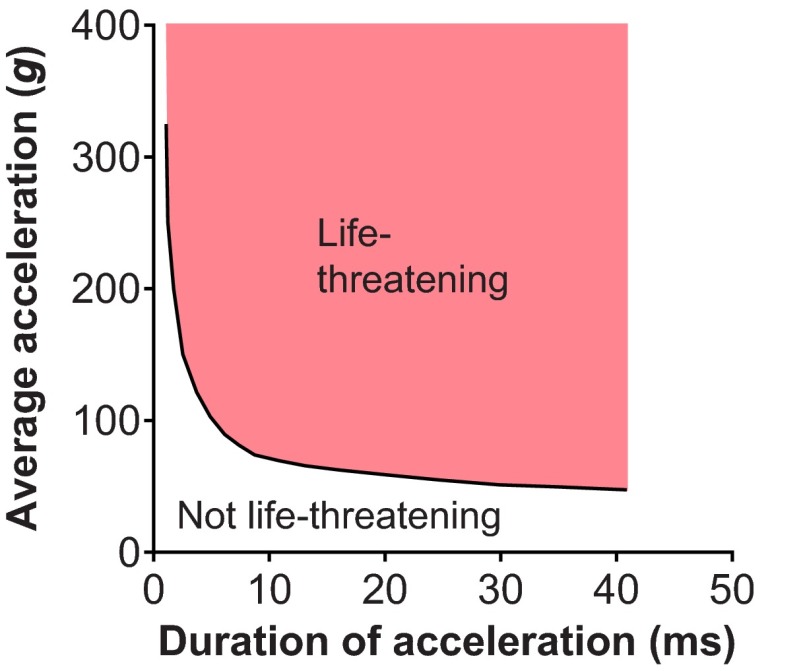
**Wayne State Tolerance Curve (WSTC).** The WSTC describes the relationship between linear head acceleration, duration of acceleration and onset of concussion (any point above the line lies above the tolerance level and could be life-threatening). The WSTC suggests that the head can withstand very high acceleration for a very short duration. Conversely, any increase in the duration of impact for the same intensity of acceleration is likely to cause head injury. Adapted with permission (Gurdjian et al., 1966) and outside the scope of the CC-BY license.

Using animal models in the 1950s, researchers at Wayne State University demonstrated that both the intensity and the duration of intracranial pressure imparted to the animal’s head (by blowing air on the exposed dura) were important to establish injury tolerance. If the time duration of exposure was very short, higher pressures could be tolerated without injury, whereas longer durations of intracranial pressure exposure caused injury at lower pressures (Gurdjian et al., 1955; Lissner et al., 1960). More sophisticated instrumentation later allowed head acceleration at the occiput to be measured, along with changes in intracranial pressure, in whole or partial cadavers subjected to forehead impacts against automotive instrument panels, windshields and non-yielding surfaces (Evans et al., 1958; Lissner et al., 1960). These experiments led to the description of the preliminary Wayne State Tolerance Curve (WSTC) for head injury. The initial tolerance curve predicted whether head injury would occur as a function of the head impact duration and the average linear acceleration measured at the occiput. This confirmed the earlier results with intracranial pressure: that subjects could tolerate higher accelerations if the duration of the acceleration exposure was short. This initial curve was further refined through additional cadaver testing and live volunteer human experiments (Eiband, 1959; Gurdjian et al., 1961; Patrick et al., 1963). The revised WSTC (Fig. 2) assumed that the underlying experimental impacts that caused a linear skull fracture also caused a moderate to severe cerebral concussion (Gurdjian et al., 1966). The WSTC was the first experimental, biomechanics-based, quantitative human brain injury criteria and it was defined based on linear head acceleration. Rotational-acceleration-based human injury criteria did not come until later (Newman, 2002) and it is still not widely incorporated in automotive safety or collision sport helmet standards. The WSTC data provide the basis for several currently widely used injury metrics, such as the Gadd Severity Index (GSI) (Gadd, 1966) and the HIC (Versace, 1971).

Several groups have generated head injury risk curves using logistic regression models based on linear acceleration as a measure of exposure (Prasad and Mertz, 1985; Hertz, 1993; Kuppa, 2004). Prasad and Mertz defined a HIC_15_ value of 700 to represent a less than 5% risk of life-threatening brain injury, with higher values of HIC corresponding to higher risks of life-threatening injury (Prasad and Mertz, 1985). More recently, Zhang and colleagues used a validated FE human head model and predicted the maximum resultant linear acceleration at the CG of the head to be 66, 82 and 106 ***g*** for a 25, 50 and 80% probability of mTBI, respectively, whereas the maximum resultant rotational accelerations for a 25, 50 and 80% probability of mTBI are estimated at 4600, 5900 and 7900 rad/second^2^, respectively (Zhang et al., 2004). Funk and colleagues estimated a 10% risk of mTBI at 165 ***g***, a HIC of 400 and an angular head acceleration of 9000 rad/second^2^ (Funk et al., 2007). To put these numbers in context, Hubbard and McLeod measured cadaver head accelerations of between 225 and 275 ***g*** over ∼3 ms when isolated heads where dropped onto their foreheads from 376 mm (Hubbard and McLeod, 1974). Thus, we would expect a person who falls onto their forehead from this height to experience similar head acceleration.

Using helmets fitted with triaxial accelerometers, Pellman and colleagues reconstructed impacts involving National Football League players and determined that peak head accelerations in concussed players averaged 98 ***g***, whereas uninjured players sustained an average peak acceleration of 60 ***g*** (Pellman et al., 2003). The lowest measured acceleration for which a player sustained a concussion was 48 ***g***. The peak angular acceleration in concussed and uninjured players averaged 6432 rad/second^2^ and 4235 rad/second^2^, respectively. The lowest angular acceleration for which a player sustained a concussion was 2615 rad/second^2^. These data are consistent with the predictions made from FE studies.

Researchers at Virginia Polytechnic Institute and State University used instrumented helmets (helmets designed with embedded accelerometers) to record tens of thousands of head impacts in football players: injuries included 57 diagnosed concussions. Linear acceleration of 171, 192 and 214 ***g*** were identified to result in a 25, 50 and 75% risk of mTBI, respectively (Rowson and Duma, 2011). Rotational accelerations of 5821, 6383 and 6945 rad/second^2^ were associated with a 25, 50 and 75% chance of mTBI, respectively (Rowson et al., 2012). The most recent study investigated mTBI injury risk as a function of linear acceleration alone, rotational acceleration alone, and a combination of both linear and rotational acceleration using logistic regression (Rowson and Duma, 2013). The predicted mTBI risk was compared with concussions sustained and recorded in football with helmet-mounted accelerometry systems and those reconstructed with crash test dummies. All three models were found to be effective predictors of mTBI. The combined model was statistically equivalent to the model using linear acceleration alone and both of these models were better than rotational head acceleration alone. The authors argued that the combined model was preferred for future investigations because it incorporated both linear and rotational acceleration and because real-world head impacts always involve a combination of the two. It is important to have effective risk curves that include both rotational and linear acceleration, because this will allow evaluation of injury prevention devices that specifically try to control rotational or linear acceleration differentially. The relative importance of rotational versus linear acceleration is still controversial and is a very active field of research. It will be important in the future to definitively establish the importance of these parameters both to evaluate innovative sports- and transportation-related injury prevention devices, and to accurately reproduce these metrics in appropriate proportions in rodent and large-animal experiments. If this is not accomplished, the resulting biochemical and histological effects in these models could poorly represent the situation in humans.

## Towards improving the biomechanical relevance of TBI in animal models

Economic, ethical and scientific drivers have shifted the focus of preclinical TBI studies from large animals to mainly rodents, even though large animals better mimic the size and anatomy of the human brain (Denny-Brown and Russell, 1940; Gennarelli et al., 1981; Gennarelli et al., 1982; Duhaime, 2006). One important consideration for selecting an appropriate rodent TBI model is that the rodent and human brain differs considerably with respect to skull anatomy, brain mass and size, craniospinal angle, gray-to-white matter ratio, and cortical folding, all of which influence the biomechanical parameters of brain injury. For example, rotational loading cannot be linearly scaled between human and rodent TBI (Finnie, 2001). Furthermore, the long axis of the rodent brain and spinal cord are nearly linear, as opposed to perpendicular axes of the human brain and spinal cord, which causes less rotational shearing in brain and renders rodents less vulnerable to diffuse axonal damage. Although the anatomical differences between the rodent and human brain will always pose a caveat for preclinical research, rodent-based experiments are expected to form the majority of mechanism-of-action and proof-of-concept studies that can then be further validated in large-animal species. This progression will provide the greatest adherence to ethical principles and scientific feasibility.

**Fig. 3.**
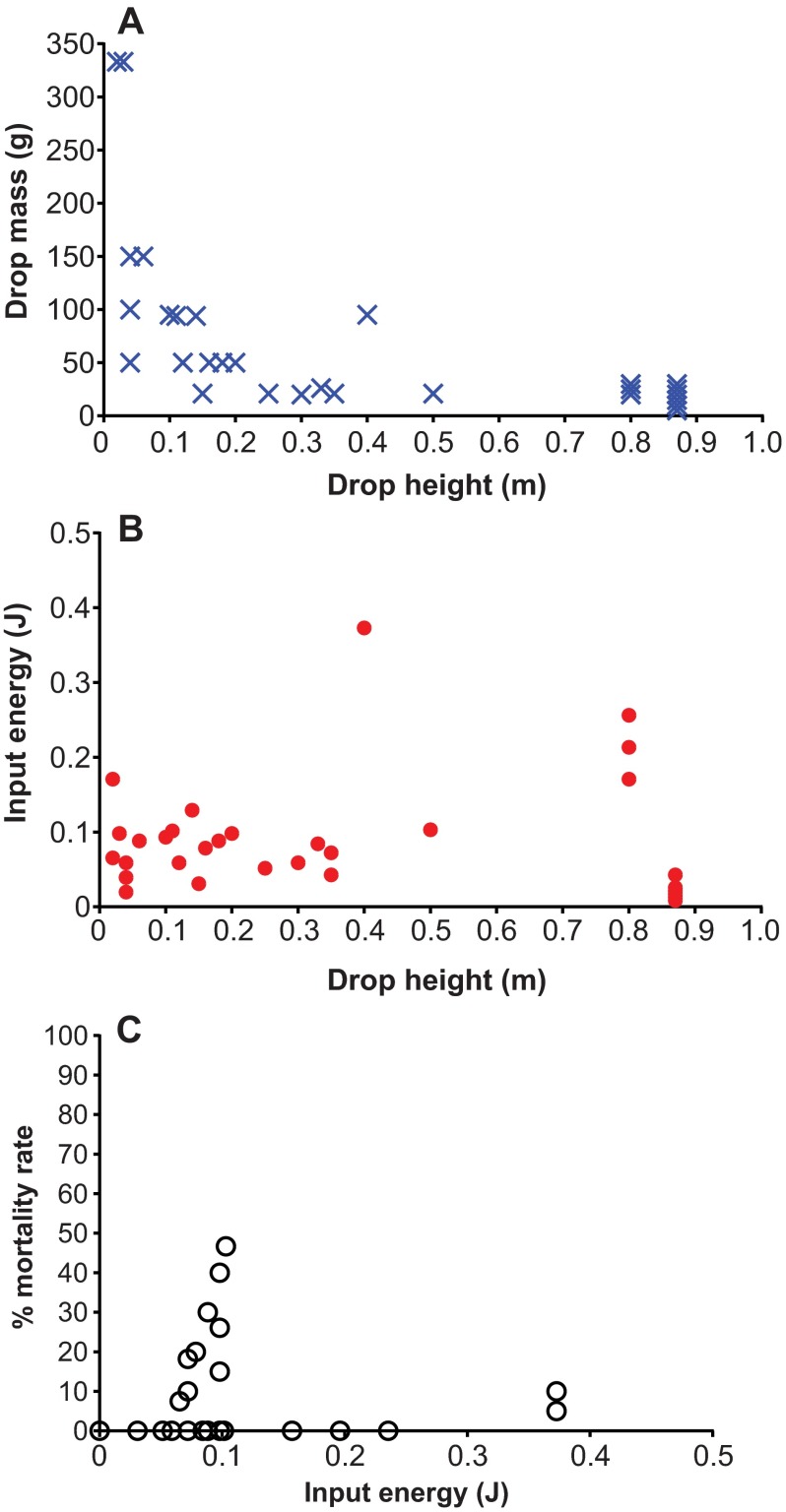
**Variability in input parameters and post-injury mortality rate in mouse weight-drop TBI studies reported in the literature.** (A) Graph illustrates variation in the two most commonly reported input parameters, drop height and drop mass. (B) Theoretical values of input energy associated with the drop height. (C) Variation in mortality rate associated with input energy is shown. Data were collected from the following studies (Manaka and Sano, 1978; Takahashi et al., 1981; Hall, 1985a; Hall, 1985b; Antal et al., 1992; Chen et al., 1996; Chen et al., 1997; Tang et al., 1997a; Tang et al., 1997b; DeFord et al., 2002; Tehranian et al., 2002; Beni-Adani et al., 2003; Pan et al., 2003; Zohar et al., 2003; Creeley et al., 2004; Milman et al., 2005; Tsenter et al., 2008; Israelsson et al., 2009; Kane et al., 2012; Zohar et al., 2011; Albert-Weißenberger et al., 2012).

**Fig. 4.**
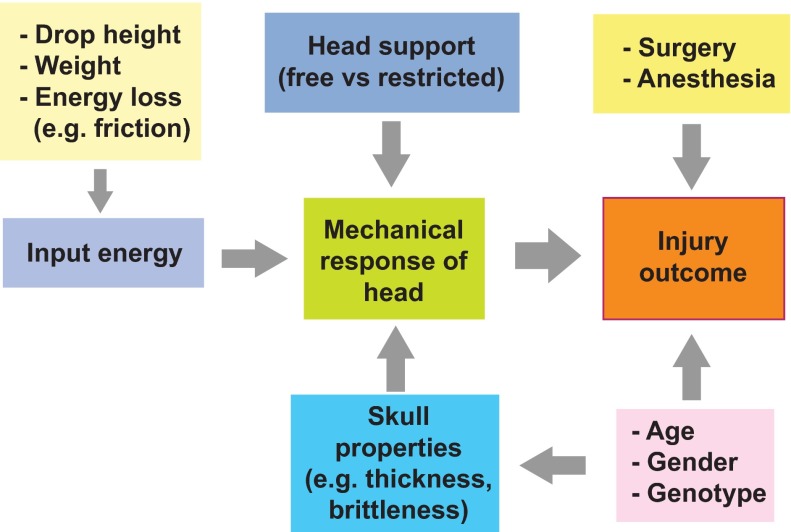
**Factors affecting standardization of rodent impact TBI models.** TBI outcomes depend on the head response to mechanical input, surgery and anesthesia, and the age, gender and genotype of animal. Head response is controlled by the input energy, head and body support (which affect movement during impact), and skull properties. The figure summarizes interactions between these experimental variables.

In contrast to FP and CCI rodent models, in which mechanical input parameters can be precisely controlled, the vast majority of rodent CHI studies lack systematic biomechanical rigor, particularly for weight-drop models, in which only gross mechanical input parameters such as the mass of the weight and drop height are typically reported. The reproducibility of these mechanical inputs and measures to decrease variability in mechanical input are rarely stated, and the mechanical response of the animal’s head to the forces applied is rarely recorded. As a result, the outcomes of studies even with similar reported mechanical inputs can vary from no detectable injury to death (Fig. 3). Important variables that could be used to improve standardization of CHI models include the reproducibility and intensity of mechanical inputs, construction of the CHI device, location and direction of impact, and quantification of animal head kinematics and injury outcomes (Fig. 4).

One potential source of variability that is particularly relevant to CHI models is the relationship of head kinematics to both behavioral and pathological outcomes after injury. A few recent studies have begun to report details of head kinematics following impact CHI in rats (Viano et al., 2009; Li et al., 2011; Viano et al., 2012). For example, Li et al. recently studied the biomechanical parameters of the weight-drop-based Marmarou impact acceleration model (Marmarou et al., 1994; Li et al., 2011). Head acceleration, change in head velocity, and linear and angular head acceleration were measured and compared with (1) kinematic responses predicted by an FE model and (2) axonal damage as assessed by APP histochemistry. The authors reported that the severity of DAI was related to the linear and angular response of the rat head but, unexpectedly, not with the drop height. Viano and colleagues developed a rat model of CHI with unrestricted head movement to mimic concussions experienced by professional football players (Viano et al., 2009; Viano et al., 2012). This important advance will make it possible to correlate behavioral and neuropathological outcomes with the biomechanical responses of an unrestricted rat head to impact. Because this information is crucial with respect to scaling CHI studies in rat to human concussion, the Viano model will offer new insights into factors that might increase the translational potential of rodent CHI studies.

Classifying injury severity in rodents depends on neurological and biochemical effects that develop over hours to weeks. Importantly, these outcomes can vary widely among different research groups. For example, motor impairment has been described as a feature of ‘mild’ TBI in some studies (Tang et al., 1997a; Tang et al., 1997b; DeFord et al., 2002; Pan et al., 2003; Creeley et al., 2004; Zohar et al., 2011) but not in others (Tang et al., 1997a; Tsenter et al., 2008). The magnitude, temporal response and signature of secondary injury response pathways can also vary among rodent species and strains. Because the severity of primary injury for mTBI can be difficult to routinely assess in most existing CHI model systems even using methods such as 7T MRI, systems that allow evaluation of the biomechanical response to injury, such as head kinematics, and recording of input injury parameters are encouraged. A better understanding of the relationships between biomechanical injury parameters and functional outcomes could offer a promising alternative approach to improve the predictive and translational power of preclinical TBI research in rodents.

## Conclusions and future directions

As neuroscientists and clinician researchers initiate programs to meet the growing demand to understand and effectively treat TBI, it is important to consider engineering principles in the selection of the most appropriate preclinical model to use. This might be particularly important for CHI models, which are designed to mimic most cases of human TBI. Although CHI models can be considered to be very similar to majority of human TBI, they are also among the most variable with respect to input parameters, pathological and behavioral outcomes, and reproducibility among laboratories. Future advances in measurement technology and improvements in the design of various types of impactors will undoubtedly help to improve the clinical relevance and translational potential of rodent preclinical TBI models.
